# Effects of clobazam for treatment of refractory status epilepticus

**DOI:** 10.1186/s12883-016-0724-y

**Published:** 2016-10-21

**Authors:** Dominik Madžar, Anna Geyer, Ruben U. Knappe, Stephanie Gollwitzer, Joji B. Kuramatsu, Stefan T. Gerner, Hajo M. Hamer, Hagen B. Huttner

**Affiliations:** Department of Neurology, University Hospital Erlangen, Schwabachanlage 6, 91054 Erlangen, Germany

**Keywords:** Refractory status epilepticus, Clobazam, Antiepileptic drugs, Outcome

## Abstract

**Background:**

Clobazam (CLB) is a well characterized antiepileptic drug (AED) that differs from other benzodiazepines by its basic chemical structure and pharmacodynamic properties. Only one previous study examined the efficacy of CLB as add-on therapy in refractory status epilepticus (RSE).

**Methods:**

We analyzed RSE episodes treated in our institution between 2001 and 2012. Successful treatment with CLB was scored if CLB was the last AED added to therapy before RSE termination. We assessed the differences between patients with and without CLB and correlated CLB with outcome. Among patients treated with CLB, we studied responders and non-responders and compared our CLB cohort with recently published data.

**Results:**

CLB was part of the AED regimen in 24/70 (34.3 %) RSE episodes. In six of these (25.0 %) RSE resolution was attributed to CLB. Baseline characteristics of episodes with and without CLB treatment showed no significant differences and RSE termination rates were very similar (83.3 % vs. 80.4 %). CLB was administered in clinically more complex RSE with longer RSE duration and worse outcome, but CLB was not related independently to outcome. Comparison of our results with previously published data revealed that baseline characteristics as well as CLB maintenance doses and time of treatment initiation were similar in both cohorts. CLB was less frequently the last AED added to RSE therapy in our patients indicating a lower treatment success rate than previously reported.

**Conclusions:**

CLB represents a reasonable AED and promising add-on agent for treatment of RSE. However, rates of successful CLB response were substantially lower than in a recently published study. Differing RSE characteristics and treatment strategies may account for the discrepancy between study results, as RSE etiologies and seizures types associated with unfavorable prognosis were more common in our cohort, while anesthetics tended to be less frequently applied to achieve seizure control.

## Background

Treatment of refractory status epilepticus (RSE) frequently requires application of more than three antiepileptic drugs (AEDs). As there is a limited number of intravenous AEDs, orally administered AEDs represent a therapeutic option particularly for longer RSE episodes [1–3]. Recently, clobazam (CLB) has been associated with high rates of RSE termination [4]. CLB is a well characterized benzodiazepine with a unique chemical structure and special pharmacodynamic properties. While various studies investigated the efficacy of CLB for treatment of refractory epilepsy, its potential in status epilepticus (SE) and particularly RSE remains uncertain [5]. We investigated the efficacy of CLB as add-on treatment in RSE and compared results with those of a recently published study.

## Methods

### Definition of refractory status epilepticus

SE was diagnosed on the basis of clinical and/or electroencephalographic evidence of seizure activity with a duration of at least 5 min, or of a series of seizures without interictal clinical recovery [6]. RSE was defined as SE refractory to administration of at least two properly dosed AEDs [7, 8].

### Identification of RSE episodes and inclusion/exclusion criteria

For identification of RSE episodes, we screened all consecutive patients treated for SE between January 2001 and December 2012 by analyzing our institutional databases including hospital records, clinical notes, and EEG reports. RSE due to hypoxic brain damage was excluded as it was considered an entity of its own with a particularly poor prognosis [9]. Furthermore, for reasons of comparability with previous studies on RSE, simple-partial and absence RSE episodes were excluded [7, 10].

### Collection of clinical and RSE-specific data

We obtained demographic baseline characteristics, laboratory findings on hospital admission, RSE-specific information, and data on treatment and complications. RSE duration was defined as time from the onset of seizures until termination, the latter being confirmed either electroencephalographically or based on clinical improvement arguing against ongoing seizure activity. RSE etiology was assessed according to ILAE criteria (cryptogenic, acute, remote, or progressive symptomatic) [11] and was considered potentially fatal when fulfilling the criteria proposed by Rossetti and coworkers [12]. Clinical severity was graded according to the Status Epilepticus Severity Score (STESS) and RSE episodes were dichotomized into STESS < 3 and STESS ≥ 3 [13]. For every AED administered, we recorded the date of first application, the duration of treatment, and the dosage. All AEDs used for RSE therapy (including initial benzodiazepines as well as anesthetics) were counted as contributing to the total number of AEDs. In all episodes in which patients received CLB, EEG data were reviewed and the episodes were grouped into the following categories based on the findings on the first EEG: +/− lateralized periodic discharges (LPD), +/− lateralized seizure patterns or lateralized rhythmic delta activity (RDA), +/− generalized periodic discharges (GPD), generalized seizure patterns, or generalized RDA.

### Outcome definition and assessment

The primary outcome measure was successful response to CLB, defined as “CLB responders” if CLB was the last AED added to treatment before RSE termination. The secondary outcome measure was functional outcome at last available follow-up. Functional outcome was assessed using the modified Rankin Scale (mRS). Poor outcome was defined when episodes either resulted in an mRS >2 or – in case of a premorbid mRS between 3 and 5 – if there was further functional decline, i.e. increase in the mRS.

### Statistical analysis

We compared CLB responders and non-responders, patients treated with and without CLB, and our CLB cohort with the one reported by Sivakumar et al. [4]. Statistics were performed using SPSS Statistics 20.0 (www.spss.com). P values of 0.05 or less were considered statistically significant and all tests were two-sided. For assessment of frequency distributions of categorical variables, Pearson *χ2* test, or where appropriate, Fisher’s exact tests were applied. We used the Kolmogorov-Smirnov test to distinguish between normal and non-normal data distribution. Normally distributed data were compared with the Student’s *t*-test, non-normally distributed data with the Mann-Whitney *U* test. In order to identify independent predictors of outcome, all variables significant on univariate analysis were entered into a forward inclusion multivariable logistic regression model. Parameters representing components of STESS were not added separately into the model when the overall STESS showed significant association upon univariate analysis.

## Results

We identified 71 RSE episodes in 65 patients. We excluded one episode because CLB treatment had been initiated before progression into RSE. Overall, RSE was treated with CLB in 24/70 (34.3 %) episodes. CLB was initiated after a median RSE duration of 2 days (IQR, 0–9 days) with a median maintenance dose of 20 mg/day (IQR, 14–23 mg/day) and was applied for a median of 8 days (IQR, 5–13 days). Therapy was preceded by a median of 4 (IQR, 3–6) other AEDs, while a median of 2 more AEDs (IQR, 0–4) followed CLB.

CLB was the last AED added to therapy and – according to the a priori definition – terminated RSE in 6/24 (25.0 %) episodes. Comparing CLB responders with non-responders revealed a significantly higher rate of acute symptomatic RSE etiology in non-responders, whereas essentially all basic characteristics as well as EEG features were similar among both groups (Table 1).

**Table 1:** Comparison of clobazam responders and non-responders

Variable	Treated with CLB (*n* = 24)	CLB responders (*n* = 6)	CLB non-responders (*n* = 18)	P value
Demographics				
Gender, female	16 (66.7)	3 (50.0)	13 (72.2)	0.362^b^
Age on admission, y	64 (52–72)	70 (59–71)	64 (50–72)	0.820^c^
Age ≥ 65 y	11 (45.8)	4 (66.7)	7 (38.9)	0.357^b^
Premorbid mRS	4 (1–4)	4 (4–5)	1 (1–4)	0.104^c^
RSE characteristics				
NCSE in coma	4 (16.7)	1 (16.7)	3 (16.7)	1.000^b^
Generalized convulsive SE	8 (33.3)	3 (50.0)	5 (27.8)	0.362^b^
Complex-partial SE	12 (50.0)	2 (33.3)	10 (55.6)	0.640^b^
Stuporous or comatose	16 (66.7)	4 (66.7)	12 (66.7)	1.000^b^
Acute symptomatic etiology	10 (41.7)	**0 (0)**	**10 (55.6)**	**0.024** ^b^
History of seizures	10 (41.7)	4 (66.7)	6 (33.3)	0.192^b^
Potentially fatal etiology	11 (45.8)	3 (50.0)	8 (44.4)	1.000^b^
STESS	3 (1–4)	3 (3–4)	3 (1–4)	0.537^c^
STESS ≥ 3	15 (62.5)	5 (83.3)	10 (55.6)	0.351^b^
EEG features				
Lateralized seizure patterns/RDA	9 (37.5)	2 (33.3)	7 (38.7)	1.000^b^
Lateralized periodic discharges	10 (41.7)	4 (66.7)	6 (33.3)	0.192^b^
Lateralized periodic discharges or seizure patterns/RDA	19 (79.2)	6 (100)	13 (72.2)	0.280^b^
Laboratory findings on admission				
Leukocyte count, ×10^3^/μl	8.5 (6.3–12.0)	8.5 (8.0–10.3)	8.8 (5.8–12.2)	0.914^c^
C-reactive protein, mg/l	18.0 (4.2–56.2)	3.4 (1.2–56.6)	33.0 (13.8–56.3)	0.446^c^
Hemoglobin, g/dl	11.2 (1.6)	10.9 (0.9)	11.3 (1.7)	0.622^d^
Serum sodium, mmol/l	136.5 (7.3)	136.8 (4.7)	136.4 (8.1)	0.908^d^
Treatment and complications				
Duration of RSE, d	16 (8–32)	6 (4–36)	16 (10–27)	0.820^c^
Length of hospital stay, d	29 (13–61)	14 (6–53)	29 (14–57)	0.673^c^
Mechanical ventilation	13 (54.2)	3 (50.0)	10 (55.6)	1.000^b^
Length of mechanical ventilation, d	6 (0–43)	0 (0–30)	6 (0–48)	0.770^c^
Use of anesthetics	11 (45.8)	3 (50.0)	8 (44.4)	1.000^b^
Induction of burst suppression	11 (45.8)	3 (50.0)	8 (44.4)	1.000^b^
Number of AEDs	7 (5–11)	9 (5–14)	7 (4–11)	0.537^c^
Use of vasopressors	13 (54.2)	3 (50.0)	10 (55.6)	1.000^b^
Sepsis	8 (33.3)	3 (50.0)	5 (27.8)	0.362^b^
Outcome				
RSE terminated	20 (83.3)	6 (100.0)	14 (77.8)	0.539^b^
Poor outcome at follow-up^a^	18 (78.3)	4 (80.0)	14 (77.8)	1.000^b^
In-hospital mortality	4 (16.7)	0 (0.0)	4 (22.2)	0.539^b^

Values are n (%), mean (±standard deviation) or median (interquartile range); significant (*p *< 0.05) parameters are expressed in bold

*Abbreviations*: *AED* antiepileptic drug, *CLB* clobazam, *mRS* modified Rankin Scale score, *NCSE* noncunvulsive status epilepticus, *RDA* rhythmic delta activity, *RSE* refractory status epilepticus, *SE* status epilepticus, *STESS* Status Epilepticus Severity Score

^a^Data available for 23 episodes

^b^Fisher’s exact test

^c^Mann-Whitney *U*-test

^d^Student’s *t*-test

Table 2 summarizes demographics, clinical and RSE-specific characteristics, as well as laboratory findings of episodes treated with or without CLB. RSE termination rates were not significantly different in patients treated with (20/24; 83.3 %) or without (37/46; 80.4 %) CLB. Therapy with CLB was associated with clinically more complex RSE, significantly longer RSE durations, and higher numbers of administered AEDs resulting in worse functional long-term outcome (Table 2; poor outcome CLB: 18/23 (78.3 %) vs. no CLB: 23/45 (51.1 %); *p* = 0.030). The median period from RSE resolution till last-available follow-up was 12 weeks (IQR, 6–35) and outcome was poor in 60.3 % of all episodes. Upon multivariable analysis, RSE duration (OR 1.10; 95 % CI: 1.00–1.28; *p* = 0.044), sepsis (OR 10.2; 95 % CI: 1.25–82.46; *p* = 0.030), and STESS ≥ 3 (OR 11.92; 95 % CI: 1.93–73.56; *p* = 0.008) – but not use of CLB (OR 1.80; 95 % CI: 0.35–9.13; *p* = 0.481) – were independently related to outcome.

**Table 2:** Comparison of episodes treated with and without clobazam

Variable	Total cohort (*n* = 70)	Treated with CLB (*n* = 24)	Not treated with CLB (*n* = 46)	*P* value
Demographics				
Gender, female	47 (67.1)	16 (66.7)	31 (67.4)	0.951^b^
Age on admission, y	70 (49–78)	64 (52–72)	73 (45–80)	0.225^d^
Age ≥ 65 y	41 (58.6)	11 (45.8)	30 (65.2)	0.118^b^
Premorbid mRS	3 (1–4)	4 (1–4)	3 (1–4)	0.694^d^
RSE characteristics				
NCSE in coma	9 (12.9)	4 (16.7)	5 (10.9)	0.481^c^
Generalized convulsive SE	31 (44.3)	8 (33.3)	23 (50.0)	0.183^b^
Complex-partial SE	30 (42.9)	12 (50.0)	18 (39.1)	0.383^b^
Stuporous or comatose	44 (62.9)	16 (66.7)	28 (60.9)	0.634^b^
Acute symptomatic etiology	31 (44.3)	10 (41.7)	21 (45.7)	0.750^b^
History of seizures	37 (52.9)	10 (41.7)	27 (58.7)	0.175^b^
Potentially fatal etiology	31 (44.3)	11 (45.8)	20 (43.5)	0.851^b^
STESS	3 (2–4)	3 (1–4)	3 (2–4)	0.550^d^
STESS ≥ 3	42 (60.0)	15 (62.5)	27 (58.7)	0.758^b^
Laboratory findings on admission				
Leukocyte count, ×10^3^/μl	9.4 (7.4–12.5)	8.5 (6.3–12.0)	10.1 (7.8–12.9)	0.197^d^
C-reactive protein, mg/l	15.7 (3.6–53.0)	18.0 (4.2–56.2)	8.3 (3.2–40.3)	0.273^d^
Hemoglobin, g/dl	11.7 (1.7)	11.2 (1.6)	11.9 (1.8)	0.110^e^
Serum sodium, mmol/l	136.9 (7.2)	136.5 (7.3)	137.2 (7.3)	0.704^e^
Treatment and complications				
Duration of RSE, d	9 (4–17)	**16 (8–32)**	**8 (4–14)**	**0.003** ^d^
Length of hospital stay, d	18 (10–30)	*29 (13*–*61)*	*18 (10*–*29)*	*0.071* ^d^
Mechanical ventilation	44 (62.9)	13 (54.2)	31 (67.4)	0.277^b^
Length of mechanical ventilation, d	3 (0–16)	6 (0–43)	6 (0–15)	0.459^d^
Use of anesthetics	43 (61.4)	*11 (45.8)*	*32 (69.6)*	*0.053* ^b^
Induction of burst suppression	28 (40.0)	11 (45.8)	17 (37.0)	0.472^b^
Number of AEDs	6 (5–8)	**7 (5–11)**	**6 (5–8)**	**0.028** ^d^
Use of vasopressors	40 (57.1)	13 (54.2)	27 (58.7)	0.716^b^
Sepsis	19 (27.1)	8 (33.3)	11 (23.9)	0.400^b^
Outcome				
RSE terminated	57 (81.4)	20 (83.3)	37 (80.4)	1.000^c^
Poor outcome at follow-up^a^	41 (60.3)	**18 (78.3)**	**23 (51.1)**	**0.030** ^b^
In-hospital mortality	12 (17.1)	4 (16.7)	8 (17.4)	1.000^c^

Values are n (%), mean (±standard deviation) or median (interquartile range); significant (*p* < 0.05) parameters are expressed in bold, parameters showing a statistical trend (*p* < 0.1) are expressed in italics

*Abbreviations*: *AED* antiepileptic drug, *CLB* clobazam, *mRS* modified Rankin Scale score, *NCSE* noncunvulsive status epilepticus, *RSE* refractory status epilepticus, *SE* status epilepticus, *STESS* Status Epilepticus Severity Score

^a^Data available for 68 episodes

^b^Pearson *χ2* test

^c^Fisher’s exact test

^d^Mann-Whitney *U*-test

^e^Student’s *t*-test

The comparison of our RSE patients treated with CLB with those from the study by Sivakumar and coworkers revealed no significant differences regarding patient age and duration of hospital stay. In our cohort, a history of seizures tended to be rarer and there was a trend toward less frequent use of anesthetics. CLB maintenance doses and days in RSE before initiation of CLB treatment were similar, whereas CLB was the last AED added to therapy in a significantly lower number of patients in our cohort (Table 3).

**Table 3:** Comparison of clobazam treated patients from the study by Sivakumar et al. and the present study

Variable	Sivakumar et al. (*n* = 17)	Present study (*n* = 24)	*P* value
Age on admission, y	63 (54–70)	64 (52–72)	0.466^b^
History of seizures	11 (64.7)	10 (41.7)	0.146^a^
CLB maintenance dose (mg/d)	20 (10–30)	20 (14–23)	0.763^b^
RSE duration before CLB, d	4 (2–7)	2 (0–9)	0.432^b^
CLB last AED added to therapy	**16 (94.1)**	**6 (25.0)**	**<0.001** ^a^
Use of anesthetics	12 (70.6)	11 (45.8)	0.116^a^
Length of hospital stay, d	30 (17–38)	29 (13–61)	0.672^b^

Values are n (%), or median (interquartile range); significant (*p* < 0.05) parameters are expressed in bold

*Abbreviations*: *CLB* clobazam, *RSE* refractory status epilepticus

^a^Pearson *χ2* test

^b^Mann-Whitney *U*-test

## Discussion

CLB has an interesting pharmacological profile and its role in the treatment of refractory epilepsy is increasingly studied [5]. The possibility of nasogastral tube administration with excellent bioavailability [14] makes CLB moreover an AED alternative worth considering in RSE. In this study, we analyzed CLB for RSE and aimed to compare our findings with recently published data. We observed less convincing results regarding CLB efficacy in seizure termination. As a consequence of different definitions of CLB treatment success, however, comparing our results with those of Sivakumar and coworkers is difficult. In their study, the latter used a strict definition only attributing RSE termination to CLB when SE was terminated within 24 h of CLB administration without changes in concurrent AEDs. Given that not all of our patients were monitored with continuous EEG and that we felt we could not with certainty exclude modifications in co-administered AEDs in a retrospective approach, we chose to use a surrogate for successful CLB treatment, i.e. whether CLB was the last AED added to therapy before RSE termination. Sivakumar and coworkers reported that CLB was the last AED added in the vast majority of their cases. Compared to our findings, this would equal a significantly higher rate of successful CLB treatment according to our definition (94.1 % vs. 25.0 %; *p* < 0.001) [4]. As dosing and timing of CLB were similar in both studies, factors not directly associated with CLB treatment seem to substantially contribute to the discrepancies between study findings. A history of seizures, for instance, tended to be rarer in our cohort. As we furthermore excluded simple-partial RSE from analysis, seizure types and RSE etiologies generally associated with a rather unfavorable prognosis [15] were probably more common in our study population. Despite this fact, the number of episodes treated with anesthetics tended to be lower than in the study of Sivakumar et al. which probably reflects diverse RSE treatment strategies and again underscores the difficulty of comparing the two studies.

Regarding our cohort, patients with and without CLB were similar in their baseline characteristics, but there were significant differences in RSE treatment-specific parameters such that CLB treated episodes appeared clinically more complex with longer RSE duration requiring more AEDs. Furthermore, CLB therapy was overrepresented in episodes in which the use of anesthetics was avoided or postponed, presumably to prevent negative side effects of therapeutic coma induction [16]. As far as one can conclude from existing studies, including the present one, it appears that CLB is rather applied in more severe RSE and is currently not considered an early choice AED [4]. Despite a comparatively low success rate, initiation of CLB was still linked to RSE resolution in some episodes and – in light of an internalization of GABA receptors with prolonged seizure activity – CLB appears to still have effects despite RSE refractoriness to benzodiazepines [17]. That we observed no CLB responders among patients with acute symptomatic RSE etiology may potentially suggest different pathophysiological mechanisms involved.

The retrospective nature limits the interpretation of our data and large confidence intervals reflect certain data instability. Moreover, the small number of RSE episodes treated with CLB reduces the generalizability of our findings. Not all of our patients received continuous EEG monitoring; this may cause imprecision in the assessment of time of RSE termination. Given the high median number of AEDs applied, effects of AEDs other than CLB in aborting seizures in our cohort cannot be ruled out in those cases in which CLB was the last AED added to therapy. We compared our patients with a cohort from a previous study which applied different criteria for inclusion and assessment of treatment response. The results of this analysis have therefore to be interpreted with due caution. We used the mRS for outcome assessment. Given a high median premorbid mRS score with some patients having 5 points already prior to admission, it is possible that in certain cases new deficits caused by RSE may have not been reflected adequately.

## Conclusions

Even if we were unable to confirm the high rates of RSE resolution reported, CLB remains an interesting AED with evident effects on RSE. Despite CLB representing a reasonable therapeutic option in RSE, based on present data, rapid SE termination – if necessary with the use of anesthetics – should not be delayed for treatment attempts with CLB. Further studies using stronger statistical designs are warranted to determine the clinical efficacy of CLB in SE and RSE.

## Abbreviations

AED: Antiepileptic drug
CI: Confidence interval
CLB: Clobazam
EEG: Electroencephalography
IQR: Interquartile range
mRS: Modified Rankin Scale
NCSE: Nonconvulsive status epilepticus
RSE: Refractory status epilepticus
SE: Status epilepticus
STESS: Status Epilepticus Severity Score
